# Trauma induced tissue survival in vitro with a muscle-biomaterial based osteogenic organoid system: a proof of concept study

**DOI:** 10.1186/s12896-020-0602-y

**Published:** 2020-01-31

**Authors:** Tao He, Jörg Hausdorf, Yan Chevalier, Roland M. Klar

**Affiliations:** 1 Department of Orthopedics, Physical Medicine and Rehabilitation, University Hospital of Munich (LMU), Munich, Germany; 2 Department of Orthopedics, Renji Hospital, School of Medicine, Shanghai Jiao Tong University, Shanghai, China

**Keywords:** Heterotopic implant model, In vitro, Tissue survival, Angiogenesis, Vasculogenesis, 3D printed β-TCP/HA, Pilot study, Organoid

## Abstract

**Background:**

The translation from animal research into the clinical environment remains problematic, as animal systems do not adequately replicate the human in vivo environment. Bioreactors have emerged as a good alternative that can reproduce part of the human in vivo processes at an in vitro level. However, in vitro bone formation platforms primarily utilize stem cells only, with tissue based in vitro systems remaining poorly investigated. As such, the present pilot study explored the tissue behavior and cell survival capability within a new in vitro skeletal muscle tissue-based biomaterial organoid bioreactor system to maximize future bone tissue engineering prospects.

**Results:**

Three dimensional printed β-tricalcium phosphate/hydroxyapatite devices were either wrapped in a sheet of rat muscle tissue or first implanted in a heterotopic muscle pouch that was then excised and cultured in vitro for up to 30 days. Devices wrapped in muscle tissue showed cell death by day 15. Contrarily, devices in muscle pouches showed angiogenic and limited osteogenic gene expression tendencies with consistent *TGF-ß*_*1*_, *COL4A1*, *VEGF-A*, *RUNX-2*, and *BMP-2* up-regulation, respectively. Histologically, muscle tissue degradation and fibrin release was seen being absorbed by devices acting possibly as a support for new tissue formation in the bioceramic scaffold that supports progenitor stem cell osteogenic differentiation.

**Conclusions:**

These results therefore demonstrate that the skeletal muscle pouch-based biomaterial culturing system can support tissue survival over a prolonged culture period and represents a novel organoid tissue model that with further adjustments could generate bone tissue for direct clinical transplantations.

## Background

The effective translation from in vitro to in vivo and in vivo to clinical practice remains a major challenge for tissue regenerative sciences [1–3]. Whilst experimental in vitro and in vivo investigations continue to contribute greatly to deciphering specific criteria in biological sciences, the translation from a functional model to the clinical setting takes an exuberant amount of time and consumes vast resources [4]. This is one of the reasons why bone tissue induction models are not yet used and the autogenous bone graft [5–9] remains the golden standard for bone regeneration clinically.

There is a clear need to develop more reliable ex vivo models where bioreactor platforms, simulating certain tissue types, have shown great capabilities at replicating certain in vivo environments [10, 11]. However, bioreactors remain problematic for use in forming a complex structure like bone, as there are various biochemical, cellular and mechanical requirements that need to be met to form this tissue type either ectopically or orthotopically [12–19], where vascularization and/or angiogenesis are essential components that help the tissue survive and grow [20, 21].

Most bioreactor platforms utilize stem cells on a specific biomaterial to produce a specific single cell derived tissue type [22–24], which is inadequate for bone tissue morphogenesis as various steps are required that together culminate in the formation of this tissue [25]. Additionally, cells cultured in vitro not only lose their homeostatic state through the loss of essential amino acids, that growth medium can hardly supply in a controlled and released state as in vivo tissue breakdown would [26], but also need to develop a viable extracellular matrix (ECM) environment first before they can thrive and grow [27, 28]. Hence, in vivo tissue based bone inductive studies remain to date the best models to study the effect of biomaterial behavior in vivo. As such, a tissue-based bioreactor platform [23, 29, 30] could be superior to that of a stem cell-based system as tissues possesses various biochemical building blocks and adult stem cell niches together with pre-established cell growth promoting environments that theoretically could provide a superior culturing milieu. However, the use of bone directly as a biomaterial growth environment in vitro is highly problematic, as culture medium cannot adequately diffuse across a hard tissue barrier [30]. Therefore, the present pilot study investigated the feasibility of two in vitro skeletal muscle-based biomaterial-culturing systems as this tissue, being considered among the promising candidate grafts for bone tissue engineering, allows for better nutrient flow [31–33]. The primary objective was to see if the models would support tissue survivability and growth into a custom three-dimensional (3D) printed bone inductive biomaterial [34, 35], whereas the secondary objective was to determine if any vasculo−/angiogenic morphogenesis, by monitoring transcriptional and translational markers, would take place as this is a crucial component required for nascent bone tissue formation [20, 21].

## Results

### Tissue pouch model supported superior tissue survival and transformation than tissue wrapping model in vitro

Many investigators have designed 3D osteogenic bioreactors utilizing different sources of cells and types of scaffolds [23, 36, 37]. However, the osteogenic transformation of fibrous tissue in vitro is conceived impossible owing to the lack of a blood supply [20]. This study attempted for the first time to establish a tissue-scaffold complex in vitro that would support tissue survivability ex vivo and cast light on inducing de novo bone formation over a long culturing period, and is meant to replicate the normal in vivo experimental environmental conditions of most known extra skeletal bone inductive models [12, 13, 16, 35].

**Fig. 1:**
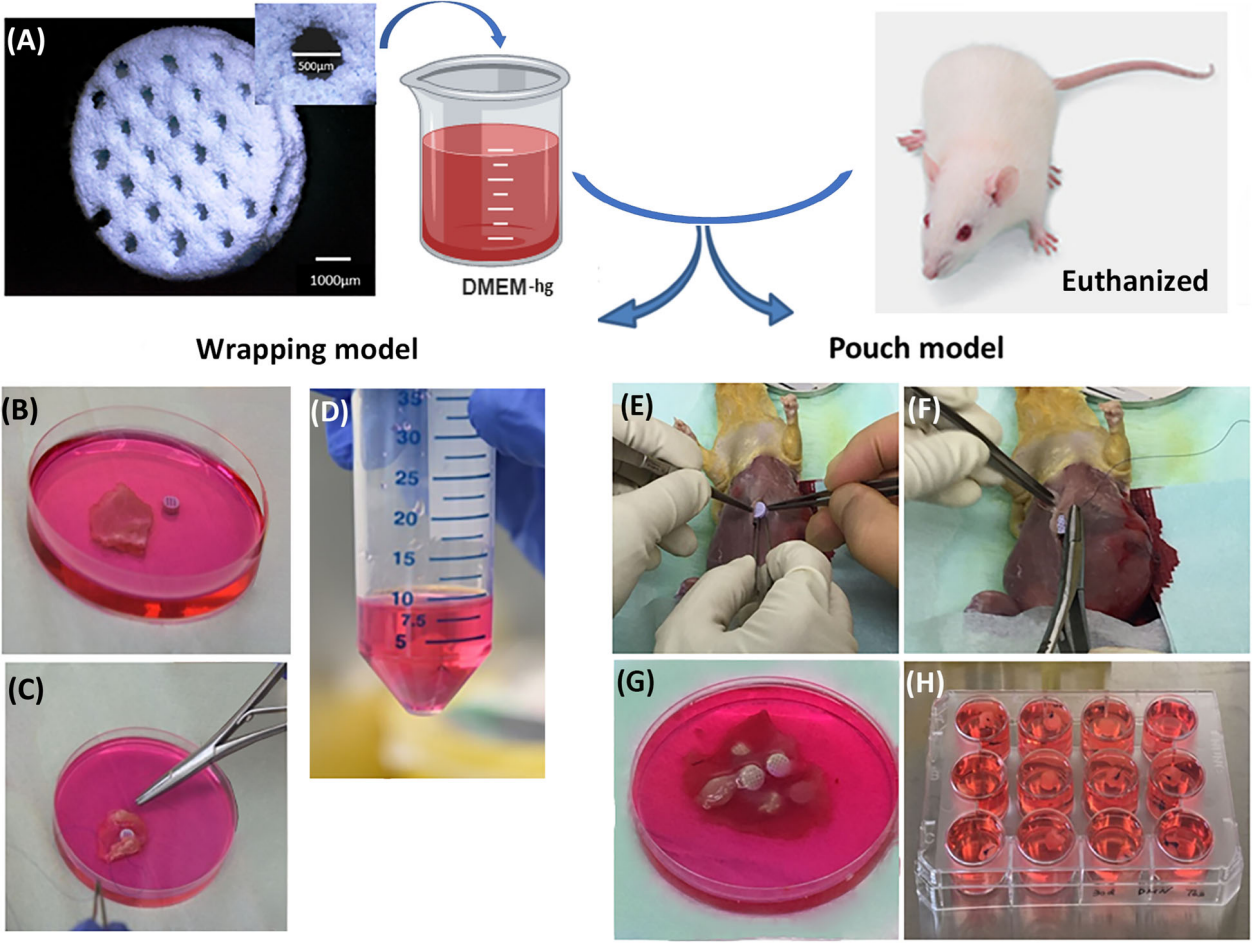
In vitro wrapping and heterotopic implanted bioceramic pouch model methodology (**a-h**). The three-dimensional printed macro-porous β-tricalcium phosphate/hydroxyapatite (β-TCP/HA) bioceramic devices (**a**), for the wrapping or pouch models, were placed in growth medium (DMEM), prior to either wrapping them in rat skeletal *rectus abdominis* muscle tissue (**b-d**), or implanting them first in heterotopic extra-skeletal *rectus abdominis* muscle sites (**e-h**) of euthanized rats. The implant sites with devices were then harvested, devices embedded in the muscle tissue excised, and subsequently placed in growth medium to be cultured for 5, 15 and 30 days in vitro. (All images within Fig. 1 originate from our own laboratory. Images were not taken from other sources)

**Fig. 2:**
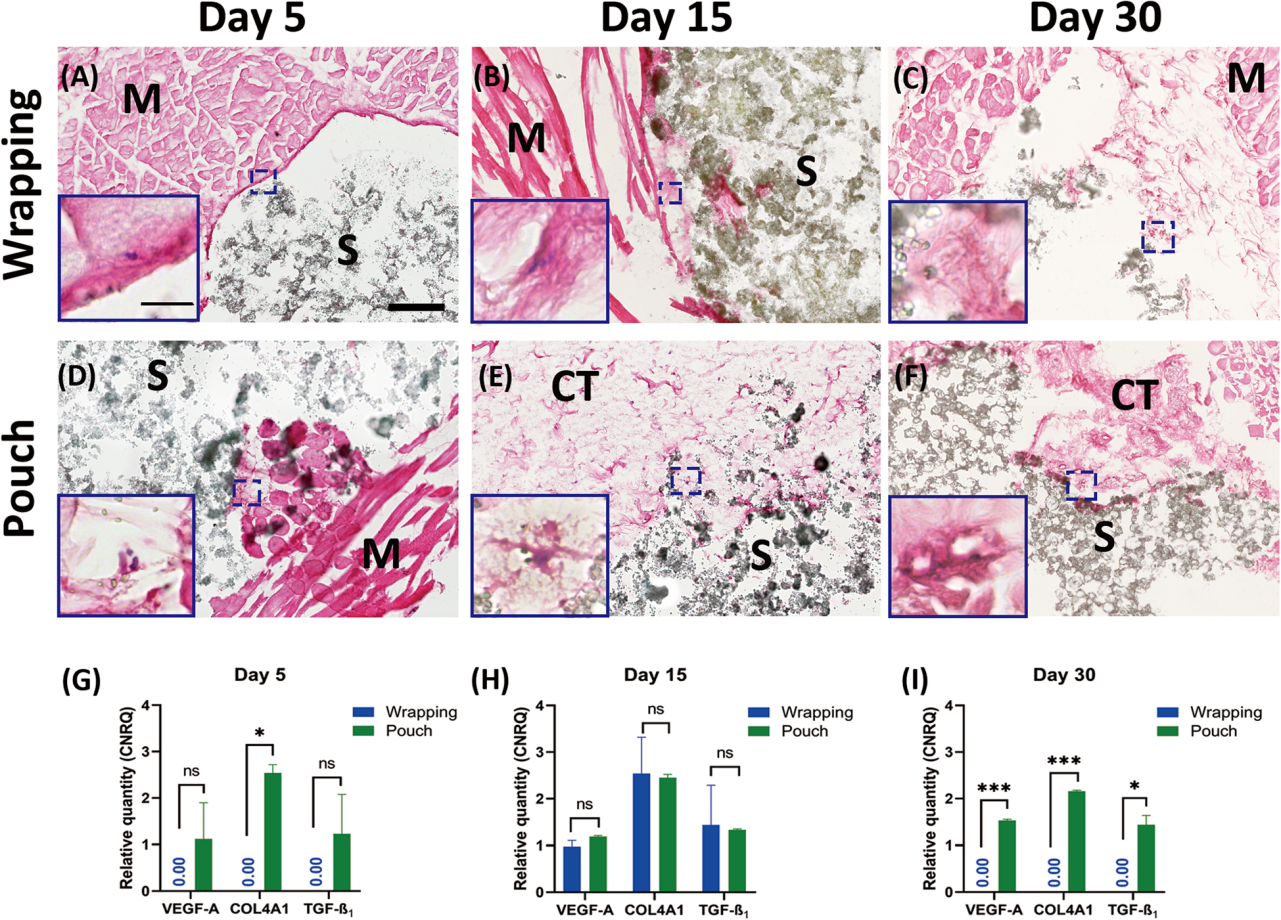
Comparison of tissue survivability between the two in vitro models in growth medium at day 5, 15 and 30 (**a-i**). Cells are confined at the interface between muscle and scaffold at day 5 in the wrapping model (**a**), with a shock silence of tissue-survival related genes (**g**). Muscle tissue undergo necrosis over time (**b**) with dying of cells (**c, i**). In pouch models, initial cell releasing occurs at day 5 (**d**), leading to successive cell migration and connective tissue formation (**e**). Vessel structures (**f**, higher power view) are still present by day 30 in vitro culturing with consistent tissue survival and growth gene expression pattern. Gene expression assays show better tissue survival in the pouch model, especially at day 30 (**g-i**). Error bars are Mean ± SEM. Ns, non-statistically significant; *, *P* < 0.05; ***, *P* < 0.001. H&E staining. M = Skeletal muscle, S = scaffold, CT = connective tissue. Bar: Lower power, 200 μm; higher power, 20 μm

**Fig. 3:**
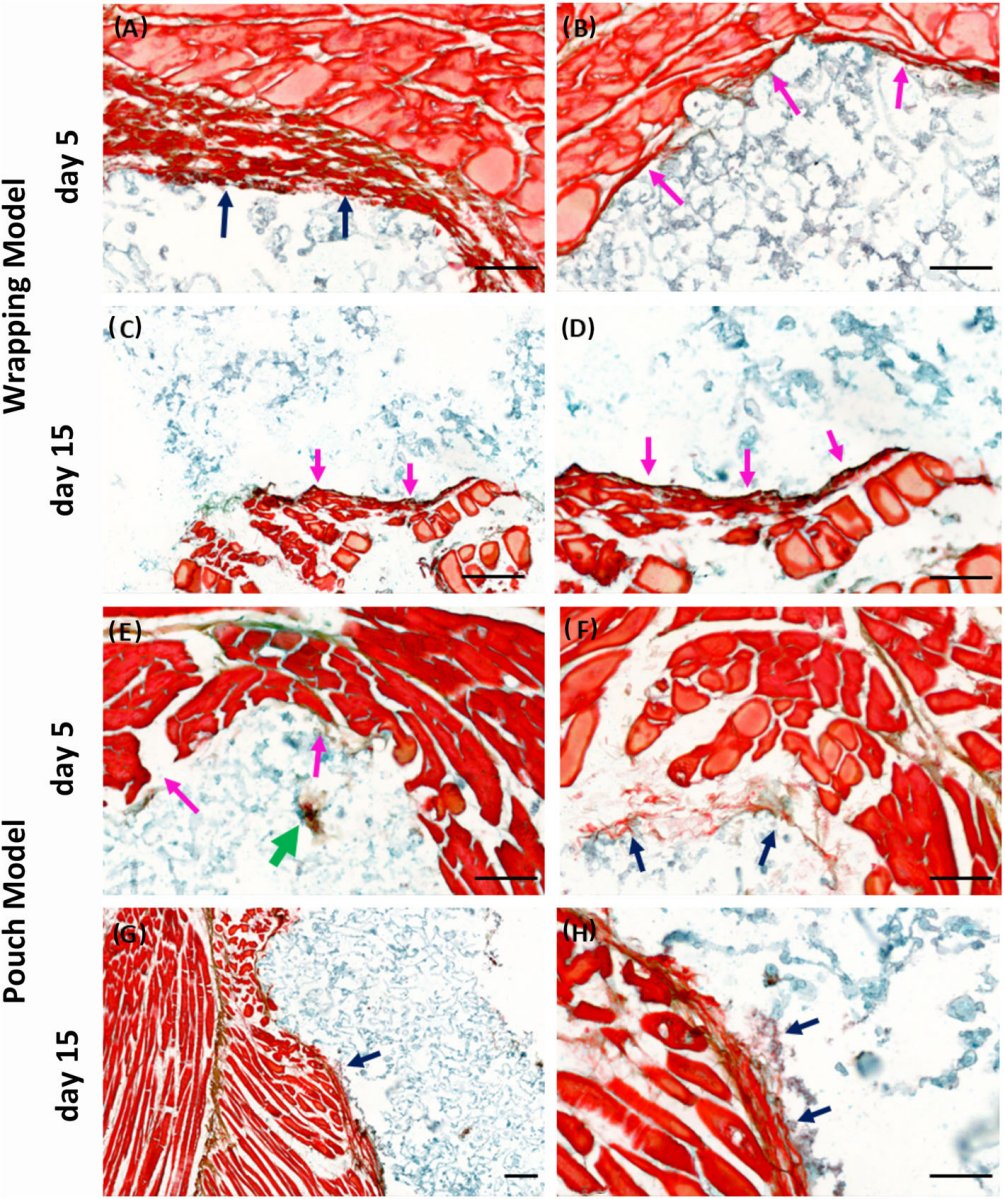
Morphology and tissue response to devices in wrapping models and pouch models at day 5 and day 15 (**a-h**). A considerable amount of fibrils were seen forming into the device (**a, f; blue arrows**) with some collagen-osteoid formation (**green arrow**) noticeable at days 5, while the self-adaptation of tissue at the periphery of device was observed in both models (**b, e; pink arrows**). In contrast, to tissue implanted heterotopically (**g, h; blue arrows**) the survivability of tissue was compromised in the tissue bag model at days 15, where the muscle tissue on the periphery of the bioceramic device was observed to undergo a type of fragmentation, discontinuing fibrous tissue formation at the interface of the muscle and device (**c, d; pink arrows**). Movat pentachrome staining was utilized to assess for collagen associated with chondrogenesis and osteogenesis, elastic fibers, muscle and connective tissue. Bars: A, B, D, E, F and H = 100 μm; C and G = 200 μm

**Fig. 4:**
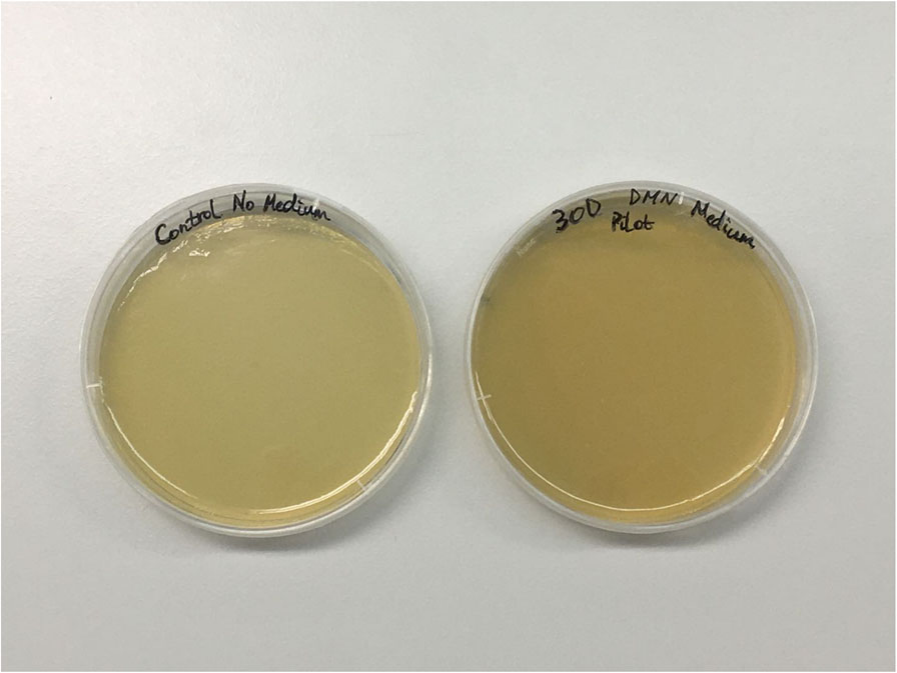
Microbiological culture results of the 30-day culturing medium with a pouch model. No microbial contamination is detected in the 30-day culturing medium with a pouch model (right plate)

**Fig. 5:**
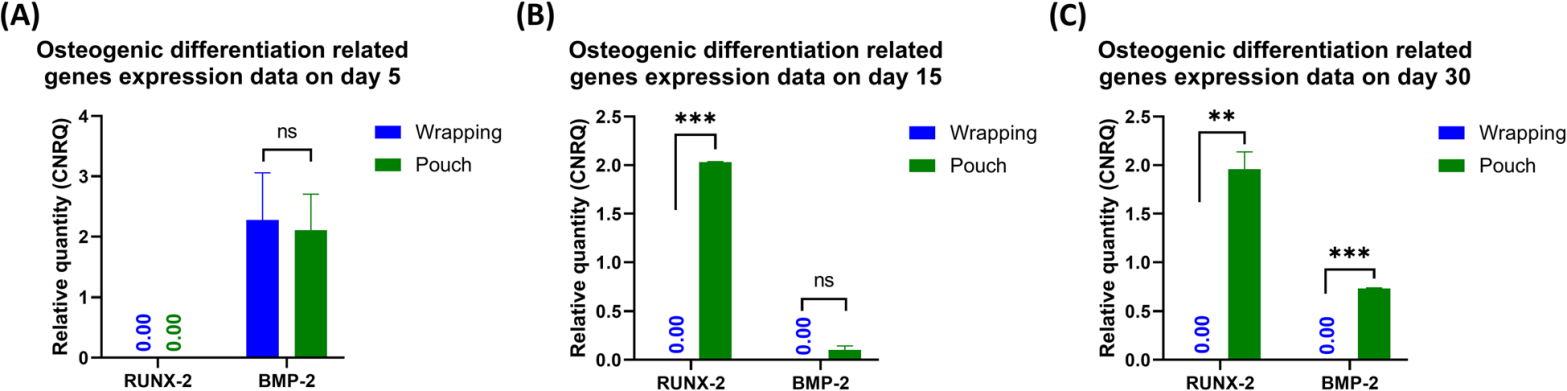
Chronological osteogenic-related gene expression pattern in both wrapping and pouch model (**a-c**). Pouch models showed superior osteogenic differentiation capacity at day 15 (**b**) and 30 (**c**) comparing wrapping models. Error bars are Mean ± SEM. Ns, non-statistically significant; **, *P* < 0.01; ***, *P* < 0.001

The abdominal skeletal muscle tissue of adult male Fischer 344/DuCrl rats was utilized, where macro−/microporous β-TCP/HA were either wrapped in the tissue harvested or where β-TCP/HA devices were first implanted in non-harvested muscle pouch within heterotopic sites, the standard experimental form to test new bone induction in vivo, and then excised before being cultured in vitro (Fig. 1). Implantation duration was limited to maximum of 30 min to prevent advanced tissue decay. In order to test the cell survival capacity of these two models, we extended the culturing time up to 30 days, where no evidence, to our knowledge, has yet reported on culturing muscle tissue ex vivo for more than 30 days. The tissue thickness was ~ 1500 μm in the wrapping model and ~ 500 μm in the pouch model. In the tissue wrapping model, no gene expression data could be generated for the 30-day in vitro β-TCP/HA wrapped in skeletal muscle tissue from rats (Fig. 2i, Fig. 5c), as the tissue became necrotic, gradually losing the original tissue structure with fading of nuclei preventing successful extraction of mRNA to be available for quantitative real-time polymerase chain reactions (qRT-PCR) analysis (Fig. 2a-c, Fig. 3a-d). On the other hand, single cells on β-TCP/HA devices pouched in the skeletal muscle survived the 30-day in vitro culturing process (Fig. 2d-f, i, Fig. 3e-h), with no bacterial contamination in the culturing system (Fig. 4). Furthermore, histological analysis showed a consistent tissue survival around the scaffold in the pouch model up to 30 days, with ongoing tissue necrosis in the wrapping model over time.

With the goal of defining the difference of gene expression pattern between these two models and evaluate which method provides better tissue survival with possible osteogenic tendencies we then compared the qRT-PCR data between them. The tissue wrapping model, only at day 15 in vitro showed an up-regulation of tissue survival and angiogenesis markers including *vascular epithelial growth factor α (VEGF-A)* and *collagen type 4 subunit 1 (COL4A1)* and *transforming growth factor β1* (*TGF-β*_*1*_) (Fig. 2h), whereas β-TCP/HA bioceramics pouched in abdominal skeletal muscle tissue of rats showed a considerable increase in angiogenesis and endothelial tissue formation genes expression at all time points (Fig. 2 g-i). For osteogenic differentiation markers, only *bone morphogenetic protein 2* (*BMP-2)* up-regulation was noticed at day 5 in the wrapping model (Fig. 5a), while both *runt-related transcription factor 2 (RUNX-2)* and *BMP-2* were steadily up-regulated over time in the pouch model and expressed at higher levels than in the wrapping model at day 30 (*P* < 0.01) (Fig. 5a-c). These results suggest better tissue survival in vitro in a tissue pouch model.

### Maintenance of vascular structure and stimulation of osteogenesis in tissue pouch models

**Fig. 6:**
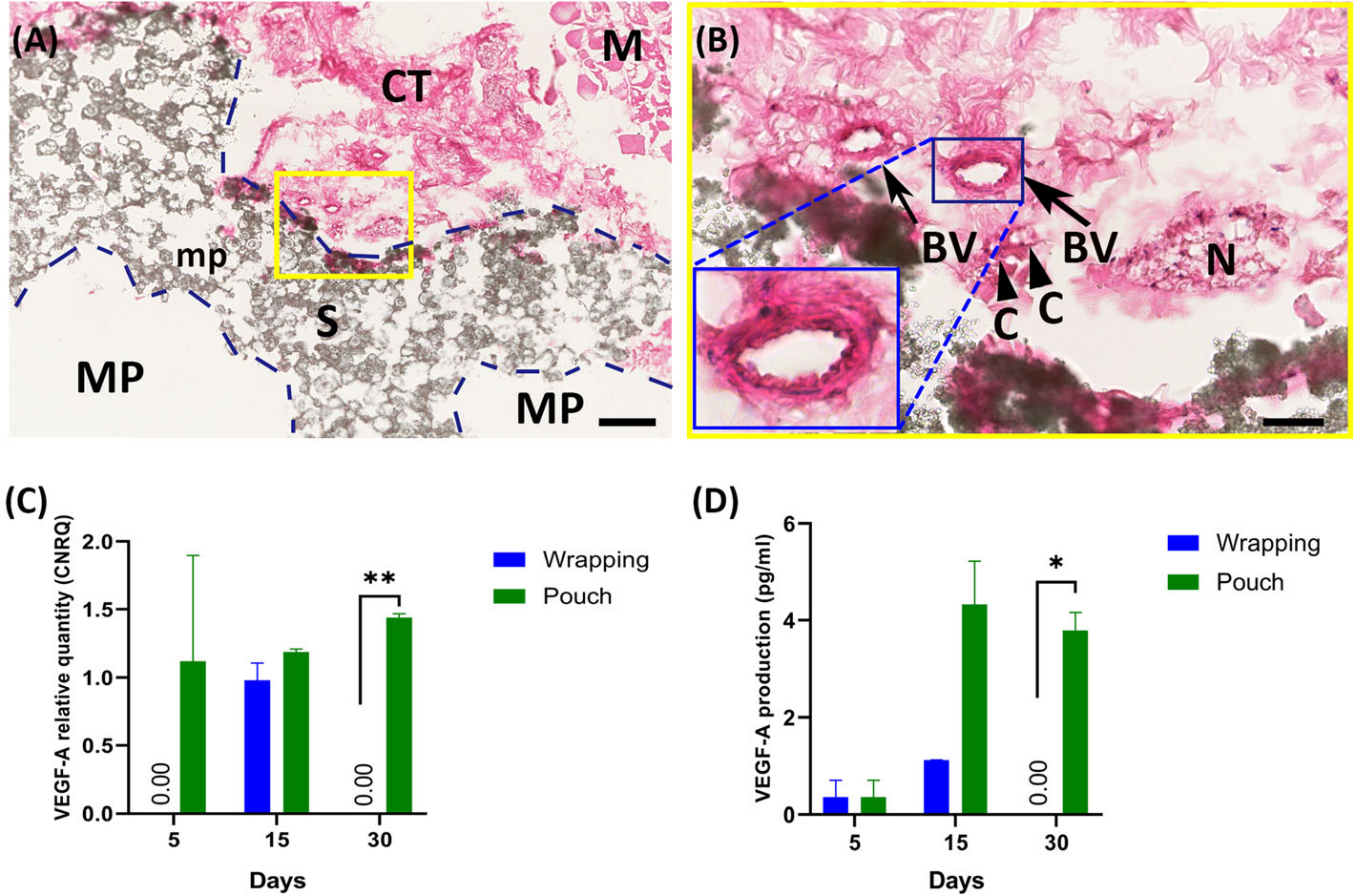
Maintenance of vascular structure and potential of angiogenesis in tissue pouch models up to 30 days (**a-d**). Connective tissue grows into the macropore of the scaffold at the periphery (**a**, dotted lines show the contour of the macropores), with neurovascular bundle still surviving by 30 days (**b**). Both transcriptional (**c**) and translational (**d**) results suggest the maintenance of angiogenesis capacity with a pouch model by 30 days, whereas the capacity is lost with a wrapping model (P < 0.01 and 0.05, respectively). Error bars are Mean ± SEM. *, *P* < 0.05; **, *P* < 0.01. H&E staining. M = Skeletal muscle, S = scaffold, CT = connective tissue, MP = macropores, mp = micropores, BV = blood vessel, N = nerve, C = capillary. Bar: A, 200 μm; B, 50 μm

Upon demonstrating better tissue survivability and growth in the tissue pouch model through histology and gene expression patterns representative of cytoproliferation and differentiation supporting new tissue formation, the chronological change of *VEGF-A* gene expression and protein production pattern up to 30 days of the culturing process and histological results at day 30 were assessed in the heterotopic pouch model (Fig. 6). This aimed to determine if a regulatory gene pattern could be identified and prove that this model indeed supports vascular structure maintenance and potential angiogenesis.

**Fig. 7:**
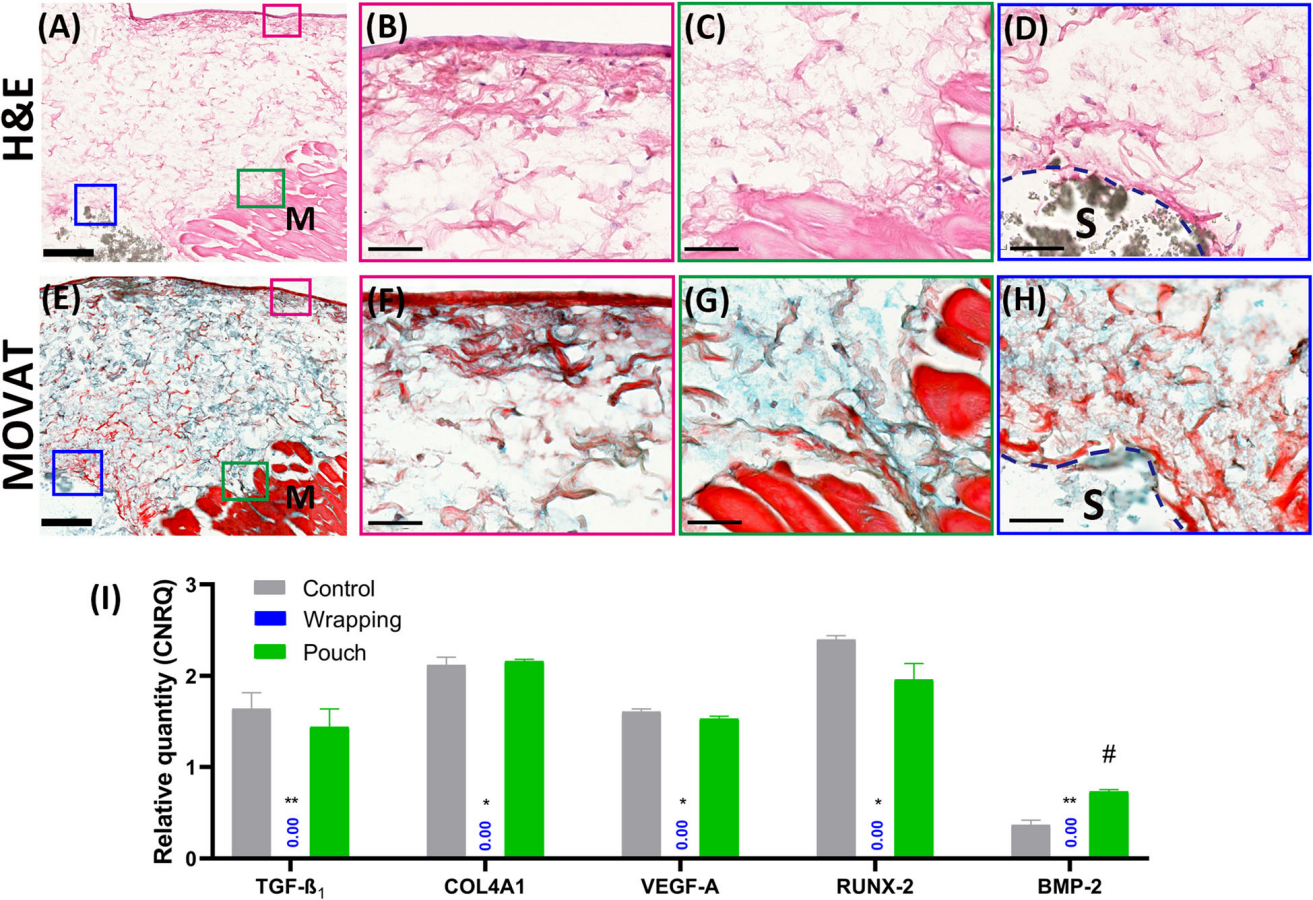
Representative morphology and tissue response to devices in pouch models at day 30 (**a-i**). Extensive connective tissue forms (**a** and **b**) around the scaffold, with comprehensive mucin deposition (**e** in blue) and fibrils (**e** in red) evenly distributed in between, consistent with the gene expression pattern showing proliferation and angiogenesis (**i**). A tissue layer forms at the interface contacting medium (**b** and **f**), where fibrous-like cells line at the surface of tissue (**b**), producing condensed fibers (**f** in red) underneath. Cells releasing from muscle fiber (**c**) migrate within the mucin-fibril rich extracellular matrix (**g**) towards either outer layer or scaffold (**d** and **h**). The osteoid (**h,** area in scarlet) mesh at the interface (dashed lines) between tissue and scaffold indicates the osteogenic transformation of the connective tissue, which is supported by *BMP-2* gene expression results (*P* < 0.05). Error bars are Mean ± SEM. *^,#^, *P* < 0.05; **, *P* < 0.01. H&E staining (**a-d**); Movat pentachrome staining (**e-h**). M = Skeletal muscle, S = scaffold. Bar: A and E, 200 μm; B-D, F-H 50 μm

In β-TCP/HA bioceramic devices muscle pouch model, the best up-regulated genes were *COL4A1*, *VEGF-A* and *TGF-β*_*1*_ at day 15 and day 30, whilst at day 5 it was *COL4A1*, *BMP-2* and *VEGF-A* (Fig. 2, Fig. 5). In short, *COL4A1* and *VEGF-A* were highly up-regulated at all time points, whilst a marked high expression of *BMP-2* occurred at day 30 compared with muscle tissue alone (*P* < 0.05) (Fig. 7i). Our findings, in the gene expression aspect, suggest that the bioceramic devices implanted in the muscle pouch support vessel survival and potential angiogenesis when cultured under normal in vitro growth conditions with limited osteogenic tendencies present, especially 30 days after treatment.

### Tissue pouch models initiate osteogenic morphogenesis ex vivo

Histological sections of β-TCP/HA bioceramic devices wrapped in rat abdominal skeletal muscle tissue clearly showed a thin layer of fibrous-like tissue lining the interface between muscle tissue and scaffold at day 5 (Fig. 2a). In contrast, fibrils and cells were released from the injured muscle fibers and attached to the interface of the scaffold (Fig. 2d). Successively, a noticeable increase of the volume of necrotic muscle fibers was observed at day 15 in the bioceramic muscle tissue wrapped model (Fig. 2b), with limited numbers of condensed nuclei containing fibers sparsely distributed within ECM at the periphery of the devices. In contrast, muscle tissue of the bioceramic devices in the heterotopic pouch model, at day 15, appeared to actively “invade” and undergo a transformation, into connective tissue (Fig. 2e) that was clearly visible at the tissue to scaffold microporous interface and could partially be observed lining the macroporous hole-like structures of the scaffold (Fig. 2e higher magnification view). By day 30, in contrast to tissue pouched bioceramic devices, as represented in Fig. 2f, tissue survival was compromised in the wrapping model, where the muscle tissue on the periphery of the bioceramic device was observed to undergo a type of fragmentation, discontinuing fibrous tissue growth at the muscle to device interface (Fig. 2c), without any presence of living cells within the scaffold (Fig. 2c higher magnification view). Contrarily, for the 30 days heterotopic pouch model group, the muscle tissue was seen breaking down (Fig. 7c and g), yet obviously supporting connective tissue that was observed invading, although mainly at the periphery, into the macroporous superstructure of the β-TCP/HA devices (Fig. 7d and h), with fibrils also appearing to interact with the particles of the porotic bioceramic scaffold. No cells or tissues pertaining to bone formation could be visualized. Subsequently, during muscle tissue degeneration, cells within and between the muscle tissue fibers were released and appeared to be migrating into the scaffold together with the extra-cellular matrix (Fig. 7a, c and d). Certain transitional zones showed some signs of a collagen-osteoid-like matrix forming near the connective tissue to porous superstructure interphase of the device (Fig. 7e and h). These results indicated that cell migration could be initiated as early as day 5, being supported up to 30 days by connective tissue in the tissue pouch model, with limited formation of collagen-osteoid-like matrices at the peripheries of the porous device.

## Discussion

Developing a new technology that can fully replicate, synthetically, an in vivo environment in vitro, however challenging, is attractive as it would allow for more efficient testing on par with the physiological reality of the clinical setting. It is expected that such medically supportive platforms would deliver faster and superior results with reduced costs whilst allowing for more accurate prediction and therapeutic models to be developed for a clinical setting [38]. Whilst one solution to this problem has been the emergence of bioreactor platforms [10, 11, 39, 40] that have a limited capacity at replicating some in vivo processes, developing a synthetic system that can fully replicate the supra-organ of bone(s), let alone induce bone formation in vitro, with its plethora of varying proteins arranged geometrically within the 3D superstructure and assortment of cellular entities [40, 41] remains perhaps the most challenging prospect for tissue engineering regenerative sciences with only the neurological complexities of the brain surpassing this endeavor. In the hereby presented pilot study, the first nascent steps towards developing such a bone inductive/formative environmental reality in vitro was attempted. Systematic studies can be further developed and improved to produce in vitro bone formation of any skeletal bone in view of clinical applications.

The bone induction principle dictates that soluble molecular signal(s) combined with an insoluble substratum are critical for the initiation and formation of de novo bone tissue formation in vivo [13, 42]. The prerequisite, in order to facilitate proper bone formation, is an adequate vascular supply, formed either by vasculogenesis and/or angiogenesis, with vessel structures invading the macro- and microporous superstructure of a device and bringing vital stem cells, nutrients, amino acids, protein signals and other resources. This would culminate in new endothelial tissue invasion into the confines of the substratum, supplying nutrients necessary for subsequent new bone tissue formation [20, 43, 44]. However, what happens to an ex vivo tissue culture model when such prerequisites are not present. Can a tissue at all survive an extended culture period let alone support new tissue morphogenesis?

Previous research has shown that for complex tissues to properly survive in vitro they require certain conditions and special applications to survive, such as electrical or biomechanical stimulation [24]. Survival is limited, in which long term studies ultimately leads to tissue degeneration in which diffusion of nutrients and tissue building resources cannot reach the relevant cells as the vascular capillary system collapses [45]. This limits the tissue development in vitro to a critical size that permits nutrient diffusion, in particular the thicker a tissue ultimately becomes in vitro will generate survival issues. Indeed, in the present study, COL4A1 was originally chosen as it is a well-known biomarker for angiogenesis, where it is critical in the basement membrane formation of new capillaries and partially also in endothelial tissue development [14, 15, 17]. VEGF-A was included after interest was aroused at whether angiogenesis could also be developed, as it is known to support the endothelial tissue formation and act as a paracrine signaling molecule on the development and proliferation of endothelial cells [46]. Interestingly, qRT-PCR analysis and histological observations in our proof-of-concept study revealed that *COL4A1* and *VEGF-A* was only briefly up-regulated within the wrapping model at day 15, after which the tissue died off in tissue culture. On the other hand, the β-TCP/HA device pouched in abdominal skeletal muscle sites, harvested and then cultured in vitro, showed a consistent and almost regulatory pattern of endothelium proliferation and/or angiogenesis up to 30 days at either transcriptional or translational level. This could also, at least for connective and endothelial-like tissue formation and invasion into the β-TCP/HA bioceramics, were validated histologically (Fig. 6). Here new connective tissue formation was histologically apparent by day 30, invading the macroporous superstructure of the devices, near the peripheries only. This deviation between the wrapping and pouch models clearly reflects the criteria of diffusion of nutrients across certain tissue thickness in vitro. On one hand, the wrapping model with a muscle thickness of almost 1500 μm prevented proper nutrient flow to the intrinsic muscle cells near the biomaterial-cell interphase. This on the other hand was not the case for the pouch model in which the muscle tissue thickness from medium-cell and cell-biomaterial interphase was only 500 μm thick. Moreover, whilst true osteogenesis eluded our investigations, as this was not a central aim as yet at this point, the gene expression level of *RUNX-2* increased considerably at both day 15 and 30 in the pouch groups with also positive up-regulation of *BMP-2* and *TGF-β*_*1*_. This suggests that the presently utilized organoid pouch model has the potential to induce new bone formation at an in vitro cell culturing level, as it was demonstrated to do in vivo in various animal models [16, 23, 27, 47, 48]. Again, possibly because of the stem cell availability and absent tissue morphogenesis due to a lack of an active blood supply that would normally bring in extra progenitor stem cells and even monocyte/macrophages critical for osteoclastogenesis [16] bone formation was retarded. Multiple studies reported that interconnection pathways have a strong impact on new tissue development, with incomplete and undersized pore interconnection limiting efficient connective tissue infiltration and blood vessels invasion into the scaffold [34]. However, in the present study, the average diameter of the interconnection pathway was ~ 40 μm, indicating the limited capacity for sound tissue and vascular invasion. This could explain why connective tissue formation and vascular survival were only observed in this study at the peripheral macropores with diameter larger than 500 μm. This leaves new strategic avenues open to improve the responsive signals in the system. Follow-up experiments need to be considered to investigate this aspect further and see how other substrata would affect tissue morphogenesis in vitro.

However, aside from the initial validations of the in vitro organoid pouch model as a tissue model to be utilized for further investigations with good survival chances as well as partial osteogenic support combined with angiogenic responses, our study serendipitously revealed new connective tissue formation and endothelial tissue survival at the peripheral region of the heterotopic pouch implanted β-TCP/HA devices. This suggests that in vitro blood vessel had survived the long-term culture period with resident cells producing the necessary signals that are required for tissue survival with the potential to angiogenesis that could support connective tissue ingrowth into the scaffold and the subsequent osteogenic differentiation of mesenchymal stem cells located within the connective tissue (Figs. 6 and 7). We postulate that the surrounding tissue in heterotopic sites are actively engaged in the formation of specific connective and/or endothelial tissue formation rather than simply providing a signal that facilitates an immunological response or acting as a stem cell reservoir to sustain the metabolic formation of new bone by induction with an insoluble substratum [13, 16–18, 47].

Various investigations into in vitro metabolistic effects of cells removed from their natural environment and cultured within an ex vivo system clearly re-iterate that cells lose their homeostatic state where critical essential amino acid building blocks, normally available for protein synthesis, suddenly disappear. This greatly limits efficient protein translation [26], including losing critical energy production requirements to fuel necessary anabolic activities to support formation of complex ECM components [49]. Catabolic reactions using glucose, adipose tissue or proteins are a necessary requirement for the survival of any cell, let alone a tissue. In vitro systems cannot adequately replicate these reactions and might prevent cellular in vitro tissue experiments from progressing past the generally accepted 30-day culturing period limit [45, 50, 51]. After this, because of extensive proliferation of cells or tissues, the catabolic breakdown into basic components and energy might be insufficient to meet the anabolic synthetic requirements to maintain cells and/or tissues active in vitro and might therefore limit their capacity to form larger complex organs. However, in light of the histological results of the present study of the skeletal muscle pouched β-TCP/HA bioceramic devices cultured in vitro at days 15 and 30, we hypothesize that the muscle tissue rescues the catabolic and anabolic homeostasis by behaving as a catabolic reservoir that breaks down into base components. Previous research by Brand (1997), Albert (2005) including Nelson and Cox (2005), have shown that as tissue degenerates in vitro it has the potential to release glucose, proteins but also critically essential amino acid that could be utilized as energy and building blocks by resident cells to support new tissue development, as seen at the peripheries of the bioceramic to muscle tissue interphase (Fig. 7) of the present study. This would allow resident stem cells to undergo differentiation and proliferation into the macroporous spaces of the bioceramic device, depositing new endothelial tissue matrix that could support vascular structures. There the culturing medium might act as a nutrient source to more effectively transport biochemical buildings blocks and nutrients into the confines of the device, providing the means for tissue survival. This form of cell differentiation and tissue repurposing or “hypertrophic tissue transformation” needs to be further validated and elucidated if it indeed is some type of tissue “recycling” modus or is simply an artefact of deterioration. Similarly, the benefit of a tissue organoid in vitro culturing system over standard cell culture modes still needs to be assessed further. We believe that the tissue organoid model might more efficiently support critical catabolic and anabolic mechanisms and assist in more complex cytological reactions, to help form more complex organ structures.

## Conclusions

The present proof-of-concept study clearly showed that an organoid pouch model exhibits superior survivability and more consistent tissue growth in vitro compared with a wrapping model, thereby rendering the approach promising for follow-up bone inductive endeavors provided the correct material and/or signals are present to facilitate this reaction. However, whilst the in vitro tissue inductive model can support the development in part of an angiogenic response, the culturing system needs to be further supplemented and enhanced with either the relevant stem cells including monocytes/macrophages/osteoclasts uniting a synthetic circulatory system that would enable future in vitro models to function as an in vivo system would. Subsequently, differences in molecular signals between in vitro and in vivo pouch models, including macro and micro signals involved in new autogenous bone formation, still need to be determined that would enable such future models to fully replicate the in vivo environment ex vivo.

## Methods

### Aims and study design

The present pilot study explored the tissue behavior and cell survival capability within a new in vitro skeletal muscle tissue-based biomaterial organoid bioreactor.

Eighteen 3D printed β-tricalcium phosphate/hydroxyapatite devices were either wrapped in a sheet of rat *rectus abdominis* muscle tissue (*n* = 9) or first implanted in a heterotopic *rectus abdominis* muscle pouch (n = 9) that was then excised and cultured in vitro for up to 30 days. Normal *rectus abdominis* muscle tissue without implants, uncultured, served as the endogenous control to which all samples were compared to. Specimens were harvested at 5 days, 15 days and 30 days (*n* = 3 per time point), respectively and underwent qRT-PCR and histological analyses. Supernatants of tissue cultures were assayed for angiogenic/vasculogenic protein production, fresh medium was the control.

### 3D printed β-tricalcium phosphate/hydroxyapatite devices (β-TCP/HA) devices

Eighteen devices were provided by BioMed Center Innovation gGmbH (Bayreuth, Germany). According to the BioMed Center, the 3D-printed β-TCP/HA bioceramic devices (Fig. 1a) had been manufactured using a mixture of tri-calcium phosphate and hydroxyapatite powders (Merck, Kenilworth, NJ, USA)) at a ratio of 40%:60%, respectively. The mixture had previously been spray-nozzle granulated from a water-based slurry with addition of organic dispersing and binding agents using a custom spray-dryer (Trema, Kemnath) and cut off at 100 μm using a classing sieve (Retsch, Haan, Germany). The lower fraction of the granulate was coated with organic adhesion-improving agents by means of fluidized bed coating; the final printing powder had size distribution values of d10 = 34.87 μm, d50 = 61.86 μm and d90 = 93.33 μm. After mixing the powder with a combination of organic additives (trade secret), the scaffolds were then printed out in a Z310 3D-Printer (3D Systems, Rock Hill, USA) using the standard colorless ink provided with the printer. After de-powdering, the scaffolds were sintered at 1250 °C, producing a solid, organic-free, porous bioceramic device with macroscopic pore channels (670.52 +/− 97.60 μm) resulting from printing design and smaller internal pores (80.95 +/− 23.38 μm) as described above. The devices were then allowed to cool, after which they were cleaned using deionized water, packed and sterilized by vacuum pulse autoclaving.

### Skeletal muscle-based biomaterial culturing models

Commercially available, four adult male *Rattus norvegicus* Fischer 344/DuCrl rats (Charles River Laboratories, Sulzbach, Germany), were utilized in the pilot study, and equally split between the two tissue models. Animals were euthanized with an overdose of isoflurane (Abbot, Chicago, USA). This was done in accordance to the rules and regulations of the Animal Protection Laboratory Animal Regulations (2013), European Directive 2010/63/EU and approved by the Animal ethics research committee (AESC) of the Ludwig Maximillian’s University of Munich (LMU), Bavaria, Germany Tierschutzgesetz §1/§4/§17 (https://www.gesetze-im-internet.de/tierschg/TierSchG.pdf) with respect to animal usage for pure tissue or organ harvest only. No muscle tissue was purchased commercially or otherwise. All muscle tissue utilized in the study was extracted by our laboratories from commercially bought adult male *Rattus norvegicus* Fischer 344/DuCrl rats (Charles River Laboratories).

Two skeletal muscle tissue biomaterial-based models were designed and tested:

#### Tissue wrapping model

For the tissue wrapping model, *n* = 9 β-TCP/HA devices, were first immersed in normal growth medium composed of Dulbecco’s modified Eagle medium–high glucose (DMEM-hg) (Biochrom GmbH, Berlin, Germany), 40 IU/mL penicillin (Biochrom GmbH) and 40 IU/mL streptomycin (Biochrom GmbH).

Two F-344 adult male rats (Charles River Laboratories) were euthanized under sterile conditions, the *rectus abdominis* skeletal muscle tissue harvested, placed in normal DMEM-hg after which 3D printed β-TCP/HA devices were wrapped in the sheets of *rectus abdominis* muscle tissue (Fig. 1b-f). Nine β-TCP/HA devices were then wrapped with a skeletal *rectus abdominis* muscle sheet, and divided into 3 culturing periods set at 5, 15 and 30 days. Each culturing period contained 3 tissue bags. *Rectus abdominis* muscle tissue without β-TCP/HA devices was cultured in parallel to tissue bags and acted as controls. Medium was changed every 2 days. Fresh *rectus abdominis* muscle tissue was used in the normalization of qRT-PCR.

#### Tissue pouch model

Nine β-TCP/HA devices were prepared by placing them in normal growth medium as explained in the section of the tissue wrapping model. Rats were then euthanized under sterile conditions, β-TCP/HA devices were immediately implanted in intramuscular *rectus abdominis* muscle pouches created by sharp and blunt dissection (Fig. 1g-j). Once all β-TCP/HA devices had been implanted, *rectus abdominis* muscle tissue pouches with biomaterials were excised using 8 mm biopsy punches (PFM medical, Cologne, Germany). Nine *rectus abdominis* muscle pouches with β-TCP/HA were created, and divided into 3 culturing periods set at 5, 15 and 30 days. Each culturing period contained 3 tissue pouches. R*ectus abdominis* muscle tissue without β-TCP/HA devices were cultured in parallel to tissue pouches and acted as controls. Medium was changed every 2 days. Fresh *rectus abdominis* muscle tissue was used in the normalization of qRT-PCR.

After the allotted culturing period, specimens with β-TCP/HA devices were harvested and cut midways, with one-half flash frozen in liquid nitrogen for qRT-PCR assays and the other half fixed in 4% paraformaldehyde (Microcos GmbH, Garching, Germany) to be processed for histological and histomorphometric analysis.

### Bacterial contamination assay

The 30-day organoid pouch model devices, during histological analysis, were observed containing a filamentous fibrous-like material. To exclude the likelihood that the fibrous like material was not of bacterial origin and in fact fibrin fibrils, the medium collect for quantitative protein analysis from these samples was subjected to a bacterial contamination test. Under sterile conditions collected culture medium was plated out on a standard Luria Broth Agar (LA) plates (1 g Tryptone, 1.5 g Technical agar, 0.5 g Yeast extract, 0.5 g NaCl (all (Sigma-Aldrich)) in 100 ml dH2O), with a normal LA plate with fresh DMEM-hg (Biochrom GmbH) medium set as control. After 72 h of incubation at 37 °C with 5% CO2, plates were assessed for bacterial colony formation by one blinded analyst (Yan Chevalier).

### QRT-PCR

QRT-PCR was performed to determine the relative gene expression quantity of tissue growth related genes especially angiogenesis and endothelial tissue formation genes, *VEGF-A*, *COL4A1* and *TGF-β*_*1*_ including known osteogenesis signaling and structural markers, specifically *RUNX-2* and *BMP-2*.

Specimen fragments for qRT-PCR were ground to powder in the presence of liquid Nitrogen. Total RNA was then isolated using a modified RNA Trizol extraction procedure (Chomczynski & Mackey, 1995). Briefly, 1 ml Trizol (Invitrogen, San Diego, CA, USA) was added to the powderised tissue, where through the addition of chloroform (Sigma-Aldrich) the aqueous RNA containing phase was transferred to Isopropanol (Sigma-Aldrich). RNA was then pelleted out in an overnight centrifugation step at 4 °C, which were then washed with 75% ethanol dried and resuspended in 32 μl RNase free water. The concentration of the RNA was determined using a NanoDropTMLite (Thermo Scientific, Waltham, USA) and quality assessed with a Bioanalyzer 2100 (Agilent Technologies, CA, USA). RNA integrity numbers lower than 8 were not accepted. RNA was then reverse transcribed into complementary DNA (cDNA) using the QuantiTect Reverse Transcription cDNA Synthesis Kit (Qiagen, Hilden, Germany).

QRT-PCR was then performed, in duplicate with FastStart Essential DNA Green Master (Roche, Basel, Switzerland) in a final reaction volume of 10 μl, using a LightCycler® 96 thermocycler (Roche). Each reaction contained 10 ng cDNA; 2x FastStart Essential DNA Green Master and 10 μM of each primer (Table 1). Primers were designed using Integrated DNA Technologies PrimerQuest Tool (https://eu.idtdna.com/Primerquest/Home/Index). Use of GeNorm (http://medgen.ugent.be/~jvdesomp/genorm/) established that *ribosomal protein large P0 (RPLP0), succinate dehydrogenase complex subunit A (SDHA)*, *RNA polymerase II subunit E (POLR2E)* and *TATA binding protein (TBP)* were the most appropriate internal reference genes to use in this experiment. All amplified PCR products underwent Sanger sequencing (GATC Biotech, Cologne, Germany) and were then analyzed utilizing nucleotide analysis (https://blast.ncbi.nlm.nih.gov/Blast.cgi? PAGE_TYPE = BlastSearch) to confirm that the correct sequence had been amplified. QRT-PCR thermocycling parameters included a pre-incubation of 3 min at 95 °C, followed by a three-step amplification program of 40 cycles consisting of a denaturation, annealing and extension step set at 95 °C for 10 s, 60 °C for 15 s and 72 °C for 30s, respectively. Relative gene expression was normalized against four reference genes. Gene expression from the harvested tissue/device models was normalized to the four reference genes and fresh abdominal skeletal muscle tissue using the Qbase+ software (http://www.biogazelle.com). Gene expression results were represented as mean calibrated normalized relative quantities (CNRQs) ± standard error, which reflect the log_10_ 2^-ΔΔCt^.

**Table 1:** Gene primer sequences for test and reference genes

Gene	Forward Primer (5′-3′)	Reverse Primer (5′-3′)
*VEGF-A*	CTACCAGCGCAGCTATTG	GATCCGCATGATCTGCATAG
*COL4A1*	CTGGGAATCCCGGACTT	GGGATCTCCCTTCATTCCT
*TGF-β*_*1*_	TTTAGGAAGGACCTGGGTT	ACCCACGTAGTAGACGATG
*BMP-2*	GGAAGTGGCCCACTTAGA	TCACTAGCAGTGGTCTTACC
*RUNX-2*	CCCAAGTGGCCACTTAC	CTGAGGCGGTCAGAGA
*RPLP0 (reference)*	CAACCCAGCTCTGGAGA	CAGCTGGCACCTTATTGG
*SDHA (reference)*	GCGGTATGACACCAGTTATT	CCTGGCAAGGTAAACCAG
*POLR2E (reference)*	GACCATCAAGGTGTACTGC	CAGCTCCTGCTGTAGAAAC
*TBP (reference)*	TAACCCAGAAAGTCGAAGAC	CCGTAAGGCATCATTGGA

### Histological evaluation

Specimens were fixed in 4% paraformaldehyde (Microcos GmbH) for 24 h after which they were processed for paraffin wax embedding. Prior to cutting 10 μm sections the surface of each paraffin block was decalcified [52]. In order to validate our gene expression patterns with respect to tissue survivability within the two tissue models, histological sections were stained using either the hematoxylin (Morphisto GmbH, Frankfurt, Germany) and eosin (H&E) staining [53] (Morphisto GmbH) or the Movat pentachrome staining [54] (Morphisto GmbH). Stained sections were subsequently analyzed under PreciPoint M8 microscope (PreciPoint, Freising, Germany).

### Quantitative angio−/vasculogenic protein assays

The amount of VEGF-A produced by the two bioreactors and controls were determined using Magnetic Luminex® Assays (R&D systems, Minneapolis, USA). Supernatants of tissue cultures were harvested at 5 days, 15 days and 30 days for either the wrapping model specimens or the pouch model specimens and controls. Absolute VEGF-A content in supernatants were measured according to the manufacturer’s instructions. Results were generated using xPONENT® 4.2 for MAGPIX® Software (R&D systems, Minneapolis, USA).

### Statistical analysis

Data were analyzed using GraphPad Prism v8.0.1 (GraphPad Software, San Diego, USA). The results were represented as mean ± standard error (SEM). Measurements were performed in triplicate or duplicate when no valid data could be obtained from one sample per group. The Mann-Whitney test was used to detect statistical differences with α = 0.05. Statistical significance was indicated by ns for no significance, * for *p* < 0.05, ** for *p* < 0.01 and *** for *p* < 0.001.

## Data Availability

The necessary algorithmic codes of the program GeNorm are readily available at (http://medgen.ugent.be/wjvdesomp/genorm/). All data, raw and processed, is readily available from the corresponding author on request.

## Abbreviations

3D: Three dimensional
BMP-2: Bone morphogenetic protein 2
CNRQ: Calibrated normalized relative quantities
COL4A1: Collagen type 4 subunit 1
ECM: Extracellular matrix
H & E: Hematoxylin and eosin
POLR2E: *RNA polymerase II subunit E*
QRT-PCR: Quantitative real-time polymerase chain reactions
RPLP0: Ribosomal protein large P0
RUNX-2: Runt-related transcription factor 2
SDHA: Succinate dehydrogenase complex subunit A
TBP: *TATA binding protein*
TGF- β_1_: Transforming growth factor β_1_
VEGF-A: Vascular epithelial growth factor α
